# Potential increased propofol sensitivity in cognitively impaired elderly: a controlled, double-blind study

**DOI:** 10.3389/fnagi.2024.1410181

**Published:** 2024-07-09

**Authors:** Huiting Zhuge, Yu Zhou, Yimin Qiu, Xiaojing Huang

**Affiliations:** ^1^Department of Anesthesiology, Shanghai Tenth People’s Hospital, Tongji University School of Medicine, Shanghai, China; ^2^Department of Anesthesiology, Shanghai General Hospital, Shanghai Jiao Tong University School of Medicine, Shanghai, China

**Keywords:** cognition, elderly, propofol, Montreal Cognitive Assessment, bispectral index

## Abstract

**Background:**

Cognitive impairment in the elderly may lead to potential increased sensitivity to anesthetic agents targeting receptors associated with cognition. This study aimed to explore the effect of cognitive status on propofol consumption during surgery in elderly patients.

**Methods:**

Sixty elderly patients scheduled for laparoscopic radical prostatectomy were allocated to either a cognitively normal [CogN, Montreal Cognitive Assessment (MoCA) score ≥26] or cognitively impaired (CogI, MoCA <26) group. Propofol was administered via target-controlled infusion to maintain a bispectral index (BIS) between 55–65 during surgery. Propofol consumption was recorded at three time points: T1 (abolished eyelash reflex), T2 (BIS = 50), T3 (extubation). BIS values at eyelash reflex abolition were also recorded. Postoperative MoCA, Visual Analogue Scale (VAS) scores, and remifentanil/sufentanil consumption were assessed.

**Results:**

BIS values before induction were similar between CogN and CogI groups. However, at eyelash reflex abolition, BIS was significantly higher in CogI than CogN (mean ± SD: 65.3 ± 7.2 vs. 61.1 ± 6.8, *p* = 0.031). Propofol requirement to reach BIS 50 was lower in CogI vs. CogN (1.24 ± 0.19 mg/kg vs. 1.46 ± 0.12 mg/kg, *p* = 0.003). Postoperative MoCA, VAS scores, and remifentanil/sufentanil consumption did not differ significantly between groups.

**Conclusion:**

Compared to cognitively intact elderly, those with cognitive impairment exhibited higher BIS at eyelash reflex abolition and required lower propofol doses to achieve the same BIS level, suggesting increased propofol sensitivity. Cognitive status may impact anesthetic medication requirements in the elderly.

## Introduction

With an increasing number of elderly surgical patients, it is crucial to understand age-related changes in the central nervous system and their potential influence on anesthetic management (Berger et al., 2015). Aging is an inevitable process, but considerable inter-individual variability exists in the rate of neurological aging due to distinct genetic and environmental factors (Wang et al., 2019). Notably, neuroreceptors in brain nuclei related to cognition are direct targets of general anesthetics. Whether cognitive decline impacts anesthetic drug requirements remains an understudied area, with limited evidence primarily from case reports.

Propofol, a widely used intravenous anesthetic agent, is favored for its rapid onset and stable cardiovascular profile. However, its sensitivity may be altered in patients with cognitive impairment. We hypothesized that cognitively impaired elderly patients would exhibit increased sensitivity to propofol compared to their cognitively intact counterparts. The primary outcomes measured in this study were propofol consumption and bispectral index (BIS) values at different time points during surgery. BIS, a processed electroencephalogram parameter, provides an objective measure of the hypnotic component of anesthesia (Punjasawadwong et al., 2018; Wang et al., 2022). Secondary endpoints included postoperative changes in cognitive function (Montreal Cognitive Assessment, MoCA) and pain scores (Visual Analogue Scale, VAS), as well as intraoperative consumption of other anesthetic agents.

By investigating the relationship between cognitive status and propofol sensitivity, this study aims to provide valuable insights into optimizing anesthetic management for the growing elderly surgical population. Understanding the potential impact of cognitive impairment on anesthetic requirements could enhance safety and improve clinical outcomes in this vulnerable patient group.

## Methods

This was a single-center, controlled study approved by the Ethics Committee of Shanghai General Hospital and registered with Chinese Clinical Trials.gov (ChiCTR-INR-1900023202). Written informed consent was obtained from all participants.

Sixty elderly male patients (65–85 years old, ASA I–II) scheduled for laparoscopic radical prostatectomy were included. Patients were excluded if they had neurological/psychiatric disorders, severe cardiovascular disease, diabetes, abnormal thyroid function, hepatic/renal dysfunction, alcohol abuse, regular analgesic/antidepressant use, preoperative severe anxiety/depression/insomnia, recent general anesthesia, or surgery scheduled after 6 PM.

Preoperatively, patients were assessed using the Montreal Cognitive Assessment (MoCA) and a visual analog pain scale (VAS). Based on MoCA scores, patients were divided into cognitively normal (CogN, MoCA ≥26, *n* = 30) and cognitively impaired (CogI, MoCA <26, *n* = 30) groups. Ultrasound-guided transversus abdominis plane blocks were performed with 0.375% ropivacaine before anesthesia induction. Bispectral index (BIS), hemodynamics, and neuromuscular blockade were monitored throughout the study. Propofol was administered via target-controlled infusion (TCI) using the Marsh model, with incremental increases until BIS reached 50. Remifentanil, rocuronium, and desflurane were also administered.

Data were collected at baseline (T0), eyelash reflex abolition (T1), BIS = 50 (T2), and extubation (T3). Postoperative MoCA, VAS scores, and anesthetic consumption were compared between groups.

Group sample size was calculated based on differences in propofol requirement to reach BIS 50 in a pilot study, in which the mean propofol requirement in the cognitive impairment group was 1.24 ± 0.19 mg/kg (*n* = 10) and in the control group, 1.46 ± 0.12 mg/kg (*n* = 10). The following formula: *n* = 15.7/ES2 + 1, where ES = effect size = (difference between groups)/(mean of the standard deviation between groups), with *α* = 0.05 and power = 0.8, was used to determine that the study would be adequately powered with *n* = 20 per group. Although our calculation indicated that 20 patients per group would be sufficient, we chose to include 30 patients per group to account for potential dropouts and to increase the robustness of our findings.

### Statistical analysis

Statistical analysis was performed using SPSS version 21.0. Continuous variables were analyzed using Student’s t-test and expressed as mean ± SD. Categorical data were analyzed using chi-squared or Fisher’s exact tests and expressed as counts (percentages). Normality and equal variance assumptions for Student’s t-test were checked using Shapiro–Wilk and Levene’s tests, respectively. Repeated measures ANOVA was used for analyzing VAS and MoCA scores over time. *p* < 0.05 was considered statistically significant.

## Results

**Figure 1:**
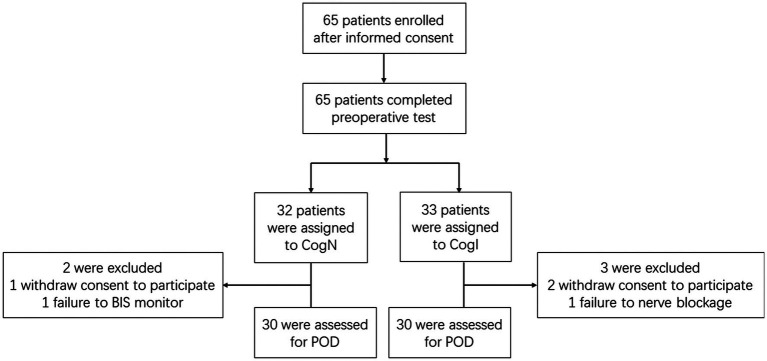
Flow diagram of participants.

Of the 65 elderly male patients screened between May 6, 2019 and March 13, 2020, 5 additional patients were excluded due to withdrawn consent, inability to assess Bispectral index (BIS) monitoring or inability to complete nerve blockage, and 60 were grouped into cognitively normal (CogN, *n* = 30) and cognitively impaired (CogI, *n* = 30) groups based on preoperative Montreal Cognitive Assessment (MoCA) scores (Figure 1). Baseline characteristics were comparable between groups, with the expected difference in MoCA scores (Table 1).

**Table 1:** Participant characteristics.

Characteristics	CogN (*n* = 30)	CogI (*n* = 30)	*p*-value
Age (year)	71.33 ± 4.76	72.33 ± 4.38	0.86
Height (cm)	169.17 ± 5.32	171.63 ± 5.02	0.86
Weight (kg)	70.57 ± 5.73	69.83 ± 6.55	0.83
Education (year)	12.20 ± 0.76	12.40 ± 1.04	0.09
ASA (I/II)	5/25	3/27	0.29
Hypertension	24 (80%)	25 (83.3%)	0.74
Coronary disease	15 (50%)	13 (43.3%)	0.61
Serum albumin	39.4 ± 2.8	39.3 ± 3.2	0.21
Montreal Cognitive Assessment (MoCA)	27.23 ± 1.01	22.43 ± 1.81	0.00
Duration of surgery (min)	164.17 ± 14.27	160.17 ± 17.15	0.66
Duration of anesthesia (min)	191.83 ± 15.67	189.00 ± 16.42	0.87
Time of extubation (min)	6.13 ± 2.01	7.50 ± 2.16	0.24

Values are presented as mean ± SD or number of patients (percentages). ASA, American Society of Anesthesiologists physical status classification.

**Figure 2:**
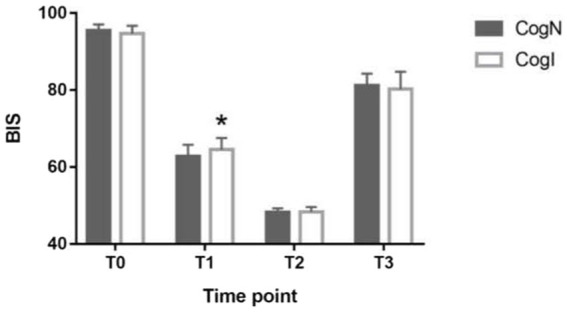
Comparison of bispectral index (BIS) at different time points. ^*^p < 0.05 vs. cognitively normal (CogN) group. T0: before induction of anesthesia; T1: abolished eyelash reflex, T2: induction to a BIS index of 50; T3; after intubation.

**Figure 3:**
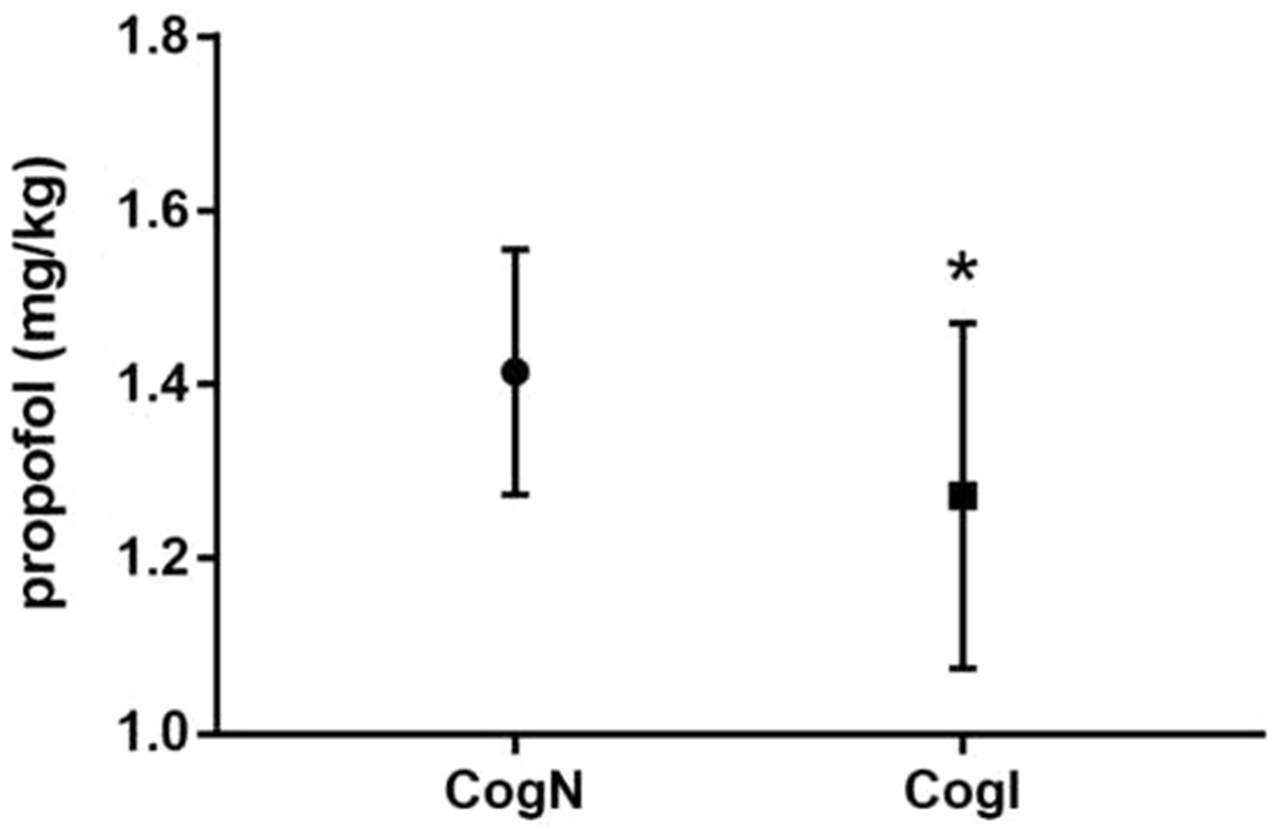
Comparison of propofol requirement at bispectral index (BIS) value of 50. ^*^p < 0.05 vs. cognitively normal (CogN) group.

BIS values at baseline and extubation timepoints did not differ significantly between CogN and CogI groups (*p* > 0.05, Figure 2). However, at the time of eyelash reflex abolition (T1), BIS was significantly higher in the CogI group compared to CogN (65.3 ± 7.2 vs. 61.1 ± 6.8, *p* = 0.031; Figure 2). The propofol dose required to reach a BIS of 50 (T2) was significantly lower in the CogI group than in CogN (1.24 ± 0.19 mg/kg vs. 1.46 ± 0.12 mg/kg, *p* = 0.003; Figure 3).

**Figure 4:**
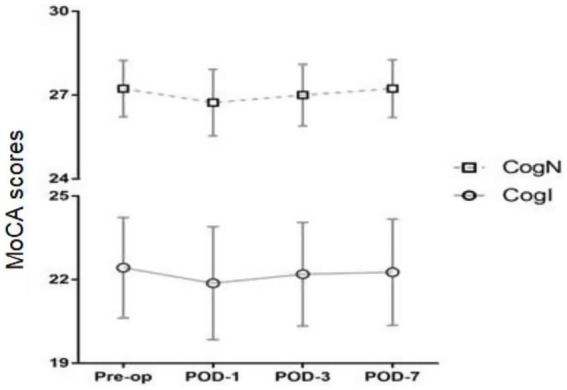
Preoperative and postoperative Montreal Cognitive Assessment (MoCA) scores, pre-op, pre-operation; POD, postoperative day.

**Figure 5:**
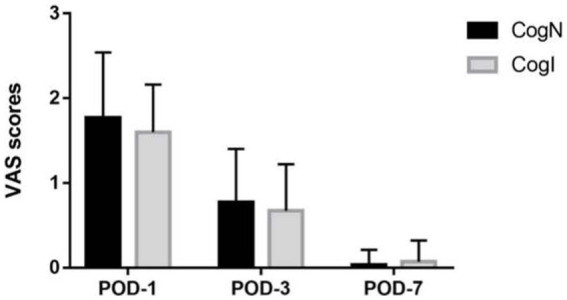
Postoperative Visual Analogue Scale (VAS) scores. POD, postoperative day.

Postoperative MoCA scores at 1, 3, and 7 days after surgery did not differ from preoperative levels in either group (Figure 4). Postoperative Visual Analogue Scale (VAS) pain scores also did not differ significantly between CogN and CogI groups at any timepoint (Figure 5).

Total intraoperative propofol, remifentanil, and sufentanil consumption were comparable between groups (Table 2). The use of vasoactive agents (atropine, ephedrine, perdipine) to manage hemodynamics did not differ between CogN and CogI groups. No hemodynamic instability or intraoperative awareness was reported in either group.

**Table 2:** Anesthetic requirement during operation.

	CogN	CogI	*p*-value
Propofol consumption (mg/kg)	2.41 ± 0.20	2.36 ± 0.27	0.39
Remifentanil consumption (μg/kg)	16.32 ± 2.85	16.36 ± 2.67	0.95
Sufentanil (mg)	18.33 ± 2.40	18.00 ± 3.11	0.64

## Discussion

The primary finding of this study was that elderly surgical patients with preoperative cognitive impairment exhibited increased sensitivity to the intravenous anesthetic agent propofol compared to their cognitively normal counterparts. This heightened propofol sensitivity manifested in two distinct ways: (1) higher bispectral index (BIS) values at the clinical endpoint of eyelash reflex abolition, indicating a deeper anesthetic level was required to achieve this milestone of unconsciousness induction, and (2) lower propofol doses needed to attain a BIS of 50, an objective measure of adequate anesthetic depth.

The lack of significant differences in postoperative outcomes despite increased propofol sensitivity during induction may be due to several factors. The use of multimodal anesthesia, including desflurane and regional nerve blocks, may have mitigated potential group differences during the maintenance phase. Additionally, our postoperative assessments may not have been sensitive enough to detect subtle cognitive changes in this population.

These results corroborate the limited prior evidence suggesting increased anesthetic sensitivity in patients with cognitive impairment and dementia (Eckenhoff et al., 2004; Bokeriia et al., 2006; Palop and Mucke, 2010; Ye et al., 2013). Mild cognitive impairment represents an intermediate transitional state along the continuum from normal aging to dementia, characterized by similar but less severe neuropathological changes such as cortical atrophy, neuronal degeneration, neurofibrillary tangles, amyloid plaque deposition, and synaptic receptor alterations (Cuninghame et al., 2023). The observed increased propofol sensitivity in our cognitively impaired elderly cohort aligns with reports of lower anesthetic requirements for induction of unconsciousness, prolonged emergence times, and exaggerated EEG suppression in patients with dementia (Pal et al., 2015; Purdon et al., 2015).

Interestingly, despite the evidence of increased propofol sensitivity during induction, we did not find significant differences in total intraoperative propofol consumption or postoperative cognitive function between cognitively normal and impaired groups. This discrepancy may relate to our anesthetic technique, which incorporated desflurane in addition to propofol for maintenance of general anesthesia. The use of this volatile anesthetic agent could have masked potential group differences in total propofol requirements during the maintenance phase. Additionally, the lack of persistent postoperative cognitive changes may reflect the acute nature of our assessments, as more long-term follow-up may be needed to detect lasting cognitive deficits in this vulnerable population (Jin et al., 2020).

The higher BIS values observed at eyelash reflex abolition in cognitively impaired patients raise important considerations regarding the accuracy and appropriate targets of processed EEG monitoring in this population. This finding could reflect true increased anesthetic sensitivity at the neurophysiological level, with a deeper degree of cerebral suppression required to reach the same clinical endpoint of unconsciousness. Alternatively, it may indicate that BIS, which is derived from frontal cortical EEG activity, does not accurately reflect the integrated anesthetic depth in patients with cognitive impairment due to underlying EEG changes associated with neurodegeneration and cortical diaschisis (Sasaguri et al., 2022).

Prior studies have suggested that reliance on EEG-based depth-of-anesthesia indices may predispose elderly patients, particularly those with cognitive impairment or dementia, to excessive anesthetic dosing beyond what is required for adequate unconsciousness and surgical tolerance (Sieber et al., 2010; Chan et al., 2013). This phenomenon could stem from the inability of processed EEG parameters to fully account for the effects of neurodegenerative changes on cerebral electrophysiology and functional connectivity across distributed neuronal networks involved in arousal and consciousness.

Our findings underscore the importance of individualized anesthetic dosing strategies tailored to the specific needs and vulnerabilities of elderly surgical patients with cognitive impairment. Excessive cerebral suppression from anesthetic overdosing can increase the risk of adverse perioperative events such as postoperative delirium, hemodynamic instability, and respiratory depression (Radtke et al., 2013; Liu et al., 2022). Conversely, insufficient anesthesia may lead to intraoperative awareness, patient movement, and exaggerated stress responses – all of which could exacerbate cognitive dysfunction in this susceptible group (Abbott and Pearse, 2019).

A balanced approach that prioritizes adequate analgesia through multimodal techniques like regional nerve blocks and local anesthetic infiltration may help reduce systemic anesthetic requirements while still providing appropriate antinociceptive coverage (Caza et al., 2008). Incorporating non-pharmacological strategies such as maintenance of normothermia, optimization of cerebral perfusion, and minimization of noxious stimuli may further mitigate the need for high anesthetic doses and their potential deleterious effects on cognition (Mason et al., 2010).

This study had several limitations, including a relatively small sample size, lack of comprehensive hematological and biochemical assessments, and inclusion of only male patients, those factors that may limit the generalizability of the findings.

We acknowledge that our reliance on a single cognitive screening tool (MoCA) is a limitation of this study. Future research would benefit from incorporating a more comprehensive cognitive assessment battery, such as the Addenbrooke’s Cognitive Examination-Revised (ACE-R) or a combination of tests targeting specific cognitive domains. This approach would provide a more nuanced understanding of the relationship between various aspects of cognitive function and anesthetic sensitivity in elderly patients.

The inclusion of only male patients limits the generalizability of our findings to the broader elderly population, as gender differences in drug metabolism and cognitive aging may influence anesthetic sensitivity.

Future studies with larger, more diverse cohorts, more extensive cognitive testing batteries, and broader anesthetic technique comparisons are needed to further elucidate optimal perioperative anesthetic management strategies for cognitively impaired elderly surgical patients.

## Conclusion

In summary, this study provides evidence that elderly patients with preoperative cognitive impairment exhibit increased sensitivity to the intravenous anesthetic propofol, as reflected by higher BIS values at unconsciousness induction and lower propofol doses required to achieve a defined anesthetic depth. These findings highlight the need for individualized anesthetic management and multimodal analgesic strategies in cognitively impaired elderly surgical patients to optimize perioperative care and mitigate potential adverse events related to excessive cerebral suppression. Continued research is needed to further elucidate optimal anesthetic approaches for this vulnerable group.

## Data availability statement

The raw data supporting the conclusions of this article will be made available by the authors, without undue reservation.

## Ethics statement

The studies involving humans were approved by Ethics Committee of Shanghai General Hospital. The studies were conducted in accordance with the local legislation and institutional requirements. The participants provided their written informed consent to participate in this study. Written informed consent was obtained from the individual(s) for the publication of any potentially identifiable images or data included in this article.

## Author contributions

HZ: Conceptualization, Data curation, Software, Writing – original draft, Writing – review & editing. YZ: Data curation, Software, Writing – review & editing. YQ: Writing – original draft. XH: Conceptualization, Data curation, Methodology, Software, Writing – original draft, Writing – review & editing.
